# A Three-Dimensional Finite Element Analysis Model for SH-SAW Torque Sensors

**DOI:** 10.3390/s19194290

**Published:** 2019-10-03

**Authors:** Chao Jiang, Yanqin Chen, Chongdu Cho

**Affiliations:** Department of Mechanical Engineering, Inha University, Incheon 22212, Korea; chiaojiang@inha.edu (C.J.); chenyanqin@inha.edu (Y.C.)

**Keywords:** torque sensing, shear horizontal surface acoustic wave (SH-SAW), quartz, three-dimensional finite element analysis (3D-FEA)

## Abstract

In this paper, a three-dimensional finite element analysis (3D-FEA) model for shear horizontal surface acoustic wave (SH-SAW) torque sensors is presented. Torque sensors play a significant role in various fields to ensure a reliable torque transmission in drivelines. Featuring the advantages of high propagation velocity, large Q-value, and good power capacity, SH-SAW-based torque sensors are promising but very few studies have been carried out. In order to develop a successful sensor, understanding the characteristics of SH-SAWs produced on piezoelectric substrates and torque sensing modes is indispensable. Therefore, in this study, we first investigated the effect on the generation of waves when different Y-cut quartz substrates are engaged. Thereafter, analyses and comparisons regarding the effect on the polarized displacement, wave guidance, and wave mode were conducted for different configurations of wave-guide layer thickness to wavelength ratios (*h_layer_/λ*) and materials. The results showed that Y-cut quartz at an angle close to 36° with a gold (Au) layer varying from *h_Au_/λ* = 0.02 to 0.03 thickness could be the most effective configuration for the excitation of SH-SAWs, compared to other combinations using platinum (Pt), titanium (Ti), and silicon dioxide (SiO_2_). Finally, based on the FEA SH-SAW torque sensor model configured with a Y + 36° quartz substrate and 0.025 *λ*-thick gold layer, the relationship between the applied torque and sensed voltage was examined, which shows a perfect linearity demonstrating the performance of the sensors.

## 1. Introduction

Ever since the first generation of surface acoustic waves (SAWs) [1] was excited by interdigital transducers (IDTs) in 1965, SAW devices have been widely investigated and used NOW for several decades [2,3,4,5,6]. They are miniature, durable, highly sensitive, reliable, and cost-effective [7]. Generally, SAW devices are used to detect Rayleigh waves [8] on piezoelectric substrates. These waves have two particle displacement components, one parallel to the propagation direction and the other one perpendicular to the substrate surface, showing an elliptical motion. Among other applications, SAW-based torque sensors are commonly applied to torque transmissions in various fields such as automobile [9,10], watercraft [11], power system [12], and industrial fabrication [5,13], which play an important role in modern society. Here, some recent literature is reviewed below.

As a pioneer in the field, Kalinin and his co-workers have made a great deal of contributions, including applications in vehicles [10], wind turbines [12], ships [14], and related interrogation techniques [10,15]. Similarly, research regarding the signal detection [16], wireless demodulation [17], and error correction [18] of SAW torque sensors has been conducted by Cai and his team. Further, in their recent article, they introduced the surface transverse wave torque sensor [19], wherein theoretical analyses on the device’s sensitivity resulting from the crystal cut were conducted, and a coupled mode approximation theory was also applied to rapidly examine the effect. However, rigorous mathematical derivations and electrical boundary conditions were simplified, resulting in large discrepancies between experimental and theoretical results. To monitor real-time torques in a rotating shaft, a system with a function of temperature compensation was fabricated by Silva et al. [5] using commercial SAW sensors. In the solution, four sensors were used: two sensors with a similar resonant frequency operating in a differential mode for torque measurement, and two others for temperature calibration. As all the components are commercial off-the-shelf, quick implementation and reliable quality are guaranteed; nonetheless, the specificity of the measurement may not be acceptable, since it is difficult to customize the size, acquisition rate, and access. In literature [20], a parameter optimization of wireless SAW torque device was presented, wherein the authors mainly concentrated on the sensor beam geometry. Essentially, this method merely reduces the negative effects of beam geometry, and the performance of SAW unit is not improved yet. 

Compared to SAW torque sensors, shear horizontal surface acoustic wave (SH-SAW) [21,22] torque sensors have scarcely been studied. Taking a look at other applications, SH-SAW-based biosensors [23,24,25] and chemical sensors [24,26] are very popular today. Since the wave’s displacement components are parallel to the substrate surface and perpendicular to the propagation direction, little damping of the wave exists in fluid or liquid, and hence, detection sensitivity can be greatly improved. In addition, SH-SAW has an approximately 0.6 times higher velocity than Rayleigh waves [27,28], thus making it possible to develop sensors that have a smaller dimension while operating at a higher resonant frequency. Furthermore, when a wireless and passive working situation is involved, the strong signal response of SH-SAWs can provide a longer interrogation distance and better detection accuracy [27]. Therefore, SH-SAW is very suitable for acoustic-based torque sensors as compared to Rayleigh wave, showing a great potential in future. However, to the authors’ best knowledge, SH-SAW has yet to be investigated for this purpose. Motivated by this, we conducted a pre-study in this study. 

In this article, a three-dimensional finite element analysis (3D-FEA) model for a guided SH-SAW torque sensor using a Y-cut quartz substrate is presented, by which the effects of crystal cuts and metal wave-guiding layers on the polarized displacements of waves, wave modes, and wave-guiding effects are analyzed and compared, providing an effective way to fully explore the interrelated impacts on the waves and devices when different configurations are assigned. Therefore, this study describes a model platform of guided SH-SAW torque sensors.

## 2. Theoretical Background

**Figure 1:**
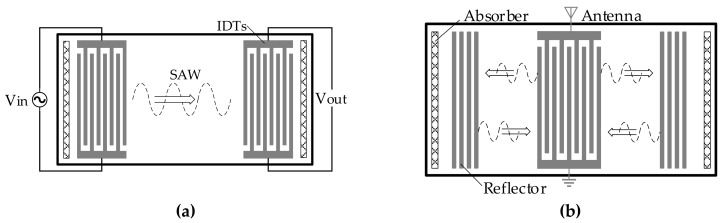
Structure of a SAW unit: (**a**) delay line; (**b**) single port resonator.

The SAW delay line or resonator is a key component for SAW sensors, which is commonly composed of a piezoelectric substrate, IDTs deposited on the substrate surface, and reflection gratings on both sides (see Figure 1). When a SAW sensor is in operation, responses arising from wave propagation perturbations caused by external disturbances can be detected, usually shown as shifting frequencies, phases, or amplitudes. By establishing an interactive relationship between response and disturbance, the physical quantity to be measured can finally be determined.

**Figure 2:**
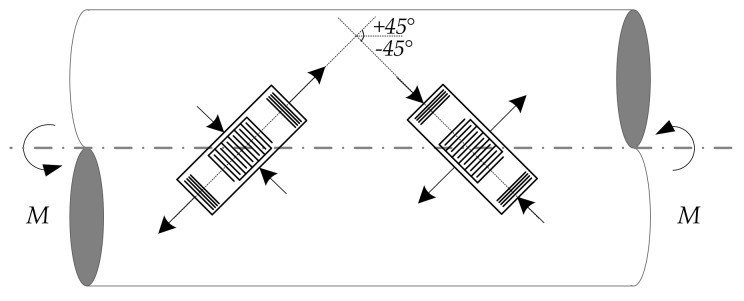
The schematic of the surface acoustic wave (SAW) torque sensor structure.

For torque measurements, two sensors with a similar resonant frequency near the industrial, scientific, and medical (ISM) radio band [5] are mounted on a shaft surface orthogonally, as shown in Figure 2, forming a ±45° angle with the shaft axis, respectively. A differential operation mode is engaged to eliminate the temperature effect. When torques are applied to the shaft’s ends, normal stresses, *σ*, oriented at the ±45° directions reach the maximum value, numerically equaling to the maximum shear stress, *τ_max_*, while the shear stresses become trivial values. Thus, the sensors undergo compression and tension only. Mathematically, the shear and the normal stress can be expressed as Equations (1) and (2), respectively:

(1) $$\tau_{\max}=\frac{16}{\pi D^{3}}M,$$

(2) $$\sigma_{\pm 45{}^{\circ}}=\pm \frac{16}{\pi D^{3}}M,$$

where *D* is the diameter of the shaft, and *M* is the applied torque. By calculating the shear stress, the applied torque can finally be solved. A good source for the relative knowledge can be found in [29]. 

To develop SAW sensors, the main determinants of wave excitation should be clearly understood, which can be summarized into three laws: (1) the piezoelectric material to be used, (2) the cut angle determining the wave propagation type, and (3) the periodicity of the IDTs [29]. In terms of SH-SAW sensors, an additional condition, a wave-guide medium using either a thin continuous film with a lower shear wave velocity or a periodic surface corrugation deposited on the substrate surface, is required, aiming to couple the wave and trap the energy near the surface [22]. The wave guided by the former medium is called the Love wave, and the other one is named as the surface transverse wave. To produce a Love wave, materials such as polymethyl methacrylate (PMMA), silicon dioxide (SiO_2_), gold (Au), and zinc oxide (ZnO) are popularly adopted [30]. Aluminum (Al) gratings and grooves created on the substrate surface are generally used [19,28] for the latter wave type. In this paper, an SH-SAW torque sensor using a wave-guide layer was investigated. 

## 3. Simulation Methodology

Tp develop a sensor, understanding the details such as its principle, sensing mechanism, and wave propagation is needed. Although many powerful techniques have been developed and successfully applied to sensor analyses, signaling mechanisms induced by internal and external factors have not yet been fully established. Analyses based on the coupled mode (COM) theory [31,32] have helped us to greatly improve our knowledge of the role of mass, temperature, and materials in SAW sensors; however, to obtain the parameters of COM, either measurement or borrowing from some related theoretical modeling such as FEA is required [33]. In the latter case, it takes a superfluous action, since FEA has already provided a way for models to quickly and easily check results as well as showing significant benefits to geometry, material, and physical field configurations [34,35]. Hence, in this research, the FEA approach was used to investigate the torque sensing mode and the effects resulting from crystal cut and wave-guide layer material and thickness.

Taking into account the merits of quartz crystal in terms of its good thermal stability and low cost for future fabrication, this material was selected as the substrate. Since SH-SAW propagates only on specific orientations [28,29], Y-cut quartz was finally specified. Instead of using a full model, a simplified 3D geometry was built to run the simulations for the sake of avoiding large memory cost and time consumption. FEA through COMSOL Multiphysics^®^ [25,34,35] was adopted in this research to facilitate computational modeling of the SH-SAW when different cut angles, wave-guide layer materials and thicknesses, and torques are involved. The piezoelectric constitutive equation [36] to be solved is expressed in Equation (3), which couples the mechanical effect as governed by Newton’s law and the electrical effect as determined by Gauss’s law:

(3) $$\begin{array}{l} T_{ij}=c_{ijkl}S_{kl}-e_{kij}E_{k}\\ D_{i}=e_{ijk}S_{jk}+\varepsilon_{ij}E_{j} \end{array},$$

where *T_ij_* is the stress matrix, *S_jk_* is the strain matrix, *E_k_* is the applied electric field, and *D_i_* is the electric displacement vector. The elastic, piezoelectric and dielectric constants are represented as *c_ijkl_*, *e_ijk_*, and *ε_ij_*, respectively. The subscripts *i*, *j*, *k*, *l* = *x*, *y*, *z*.

**Figure 3:**
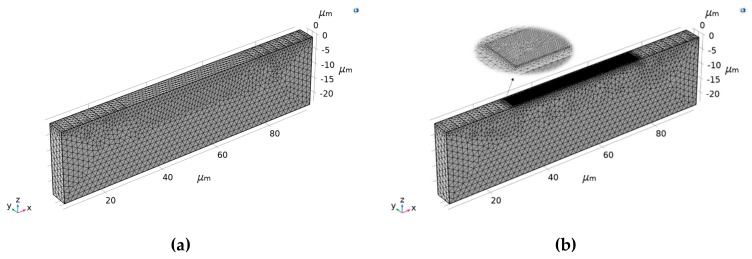
The geometry used for the three-dimensional finite element analysis (3D-FEA) simulation, (**a**) without a wave-guide layer and (**b**) with a wave-guide layer.

The 3D geometry used for the simulations is shown in Figure 3. Massless input and output IDTs of aluminum (Al) with a dimension of 0.25 *λ* width by 0.5 *λ* length are built on the left and right sides of piezoelectric substrate (8.75 *λ* × 1.5 *λ* × 2.5 *λ*), respectively. Thus, a SAW of wavelength *λ* (equaling 11.54 μm) can be generated propagating along the *X*-axis. To study the wave-guiding effect, a wave-guide layer (3.75 *λ* × 0.3 *λ* × *h_layer_*) is accordingly placed in the middle. Reflections caused by the deposited layer and IDTs are neglected, and perfectly matched layers (PMLs) [37] are used to absorb the waves at the lateral boundaries. In the electrostatics node, the even fingers of the input IDTs are connected and assigned with a +5 V voltage, while the counterparts in the output IDTs are treated as sensing ports. The rest of the IDT electrodes are grounded directly. A rotated coordinate system depicted by three Euler angles according to the ZXZ convention [38] is applied to simulate the piezoelectric substrate with the desired orientation. Different boundary conditions are assigned to the sides of the substrate for different simulations. To investigate the crystal cut effect and wave-guide effect, all the sides of the substrate are set to be free except for the bottom. On the other hand, to sense the external torques, boundary loads are exerted to the lateral faces, while the top and bottom are free and fixed, respectively. In FEA, geometries are subdivided into small elements, and dimensions of at least five times smaller than *λ* are required when it comes to acoustic wave problems [39]. Considering that the energy of the generated wave mainly concentrates at the top surface, a finer mesh is given to this boundary than in the bulk. Free triangular meshing is distributed to the surfaces, while the remainder is tetrahedral.

## 4. Results and Discussion

### 4.1. Effect of Crystal Cuts

**Figure 4:**
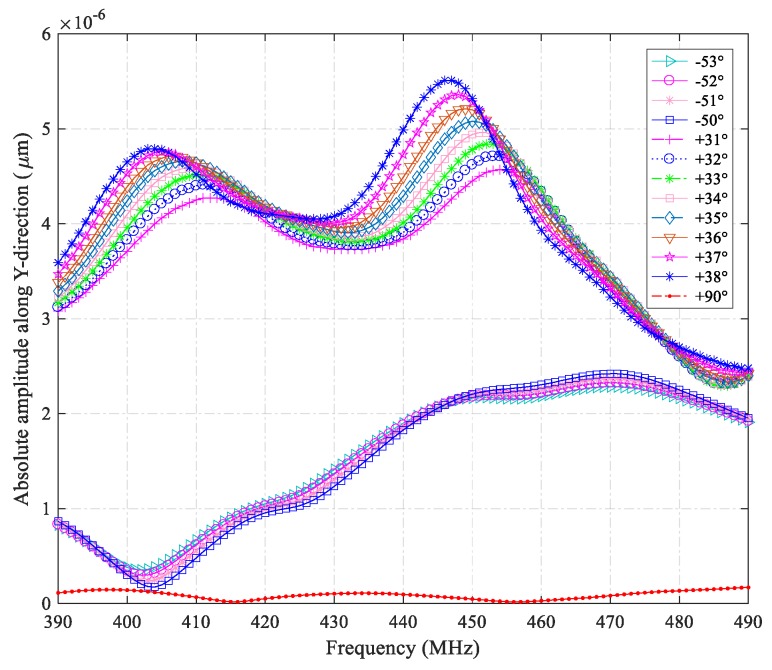
Absolute amplitudes of SH-SAWs along the Y-direction regarding different cut angles (rotations from −53° to −50° are with a five-fold offset).

A notable characteristic of SH-SAW is that the main displacement component is perpendicular to the propagation direction (*X*-axis) and parallel to the surface (XY-plane). Thus, when searching for a dominant SH-SAW mode in a cut, we search for the dominant Y- component. The result of the crystal cut effect is illustrated in Figure 4, which shows the absolute amplitude of the wave along the *Y*-axis, plotted against frequency for different cut angles including the commonly used cuts, Y + 36° and Y − 51° [19]. It was found that with the incensement of the cut angle, the curves’ peaks shift from right to left, which indicates the changing tendency of the dominant SH-SAW modes. In particular, we examined and compared the results in the crystal cuts of Y − 51°, Y + 36°, Y + 38°, and Y + 90°. At 446 MHz and 403 MHz, the strongest peaks were observed on the Y + 38° cut substrate. Checking the SH-SAWs for the two special cuts, peaks at 405 MHz and 449 MHz were found on the Y + 36° cut base, and a peak at 470 MHz on the Y − 51° cut substrate. In terms of the Y + 90° cut, the curve shows few changes in comparison with the others, which indicates that virtually zero SH-SAW components exist in the range, and there is no resonant frequency for this cut. 

**Figure 5:**
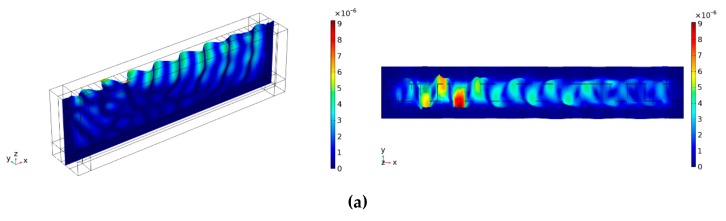

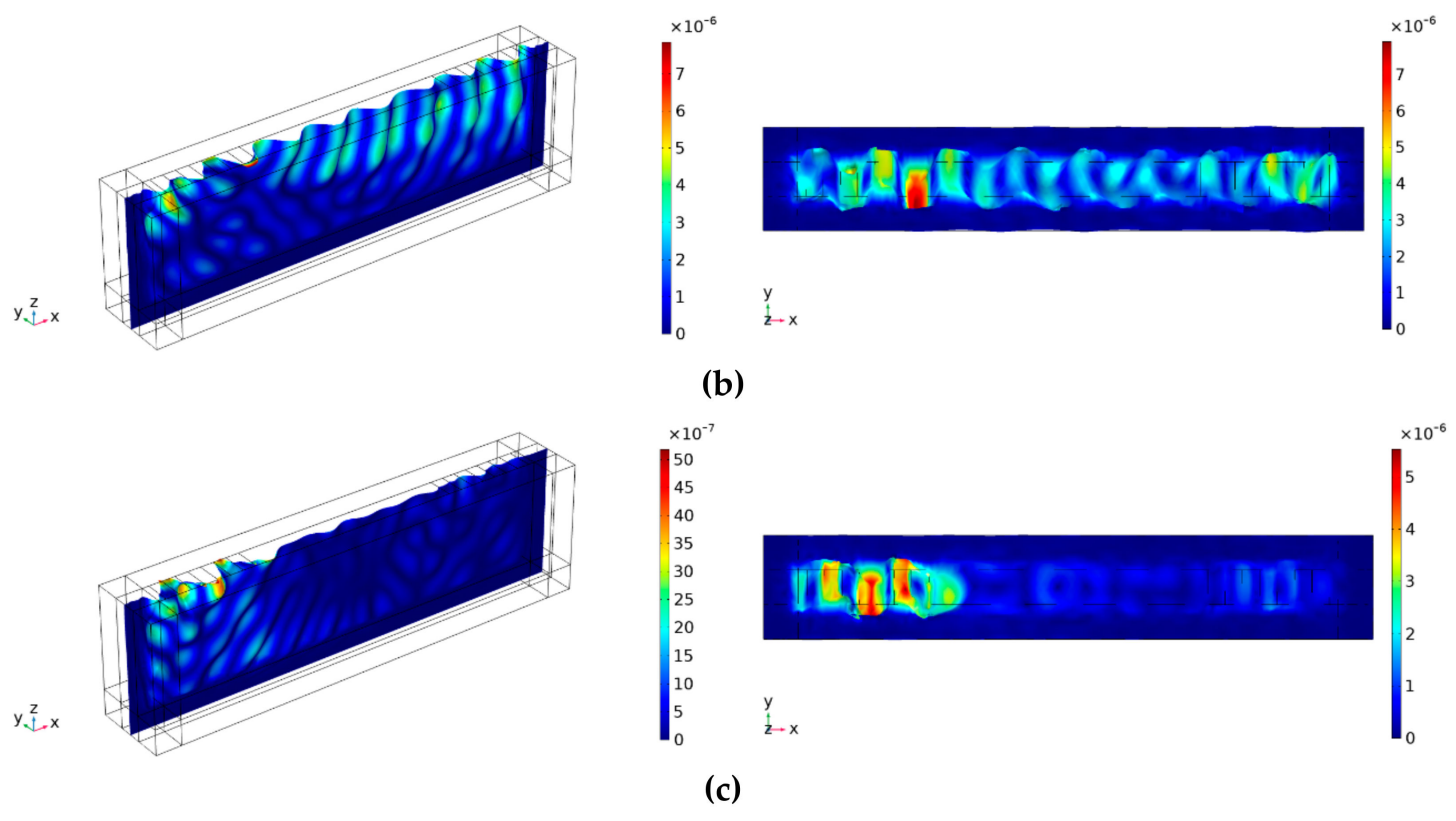
Snapshots of sectional views from different substrates: (**a**) Y + 36° cut at 407 MHz; (**b**) Y + 36° cut at 449 MHz; (**c**) Y − 51° cut at 470 MHz.

To examine the details of the waves on the Y + 36° and Y − 51° cut substrates, sectional views regarding the 3D displacements were further reviewed, as shown in Figure 5. They correspond to the main wave modes plotted in Figure 4. From the images, it can be seen directly that the waves are propagating along the *X*-axis and perpendicular to the *Y*-axis, whose displacement on the *Y*-axis is predominant among the three components showing a feature of SH-SAWs. However, as waves propagate, the wave energy gradually permeates into the bulk exhibiting a character of surface-skimming bulk wave (SSBW) [36]. It was found that the waves propagating on the Y + 36° substrate are in good agreement with a sinusoidal pattern demonstrating a periodic shear wave on the surface, while the ones on the Y − 51° substrate are very bad, where we can hardly see the propagation on the top, so it can be known that Y + 36° could be a better cut for a quartz substrate to generate SH-SAWs in the examined frequency range. 

### 4.2. Effect of Wave-Guide Layers

Based on the study above, a Y + 36° cut quartz was selected as the substrate to investigate the waveguide effect of different guiding layers. For this, material and thickness were considered as the parameters. 

In the present work, four frequently used materials were seleted on the basis of material properties and applications. Gold (Au), having the highest ductility and excellent chemical stability, is a common coating material in sensors. Platinum (Pt) is also an inert metal characterized by good chemical and thermal stabilities. Owing to a relatively low density and high shear velocity of titanium (Ti), this material was chosen as an interesting contrast. Finally, we added silicon dioxide (SiO_2_) as the fourth material due to its low price as well as good elastic, thermal property, and low acoustic loss. For convenience, Table 1 lists some important constants of the materials. Moreover, the thickness, *h_layer_*, varying from 0 to 0.035 *λ* with a 0.005 *λ* step, is set. 

**Figure 6:**
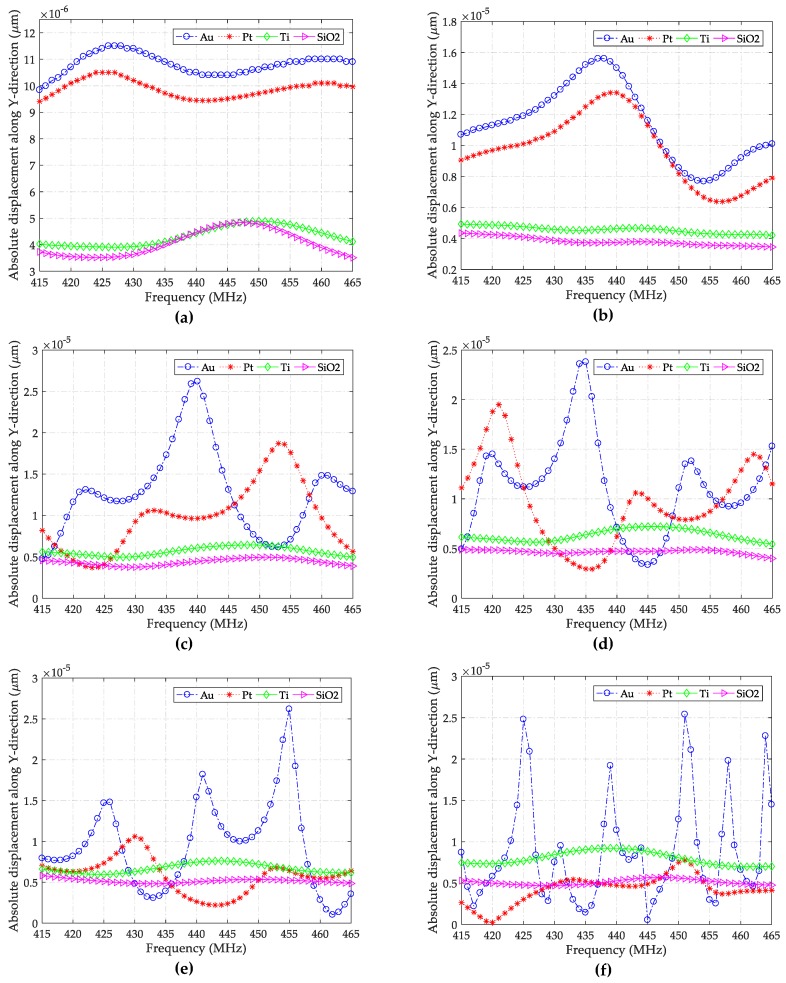
Absolute displacements of SH-SAWs in the Y-direction for various *h_layer_/λ* ratios: (**a**) 0.01; (**b**) 0.015; (**c**) 0.02; (**d**) 0.025; (**e**) 0.03; and (**f**) 0.035.

Figure 6 presents the absolute displacements of the guided waves along the *Y*-axis plotted against frequency for different wave-guide layer thicknesses and materials. It can be noticed that the number of peaks grows as the thickness of Au layer increases, indicating a growing number of wave modes [40]. Examining the corresponding amplitudes, we can see that when *h_Au_* is larger than 0.015 *λ*, the magnitudes of the strongest peaks are very similar, indicating that the thickened layer cannot further enhance the excitation and continuously increasing the thickness will make no sense. However, it is possible to see more wave modes when this parameter is further enlarged, because the number of modes in Figure 6f increases rapidly compared to those in other figures.

However, too dense modes are not beneficial to sensor design, so a gold layer with a thickness varying from 0.02 *λ* to 0.03 *λ* could be a good configuration for guiding SH-SAWs. Taking a look at the curves obtained using a Pt layer, an interesting phenomenon is observed: The number of wave modes increases first when *h_Pt_* grows from 0.01 *λ* to 0.025 *λ*, and then it decreases as the thickness increases further. A similar phenomenon also occurs with the polarized amplitudes, but faster. This means that a thin Pt layer may be sufficient to guide SH-SAWs because the number of wave modes is more susceptible to thickness than polarized displacement. With respect to the remaining two layers (Ti and SiO_2_), the presented wave modes and amplitudes remain relatively unchanged to the changing thickness and frequency, which indicates that: (1) the induced waves are insensitive to thickness variation, or (2) the layers cannot effectively guide the waves. To guide SH-SAWs, maybe, these two materials are not suitable.

**Figure 7:**
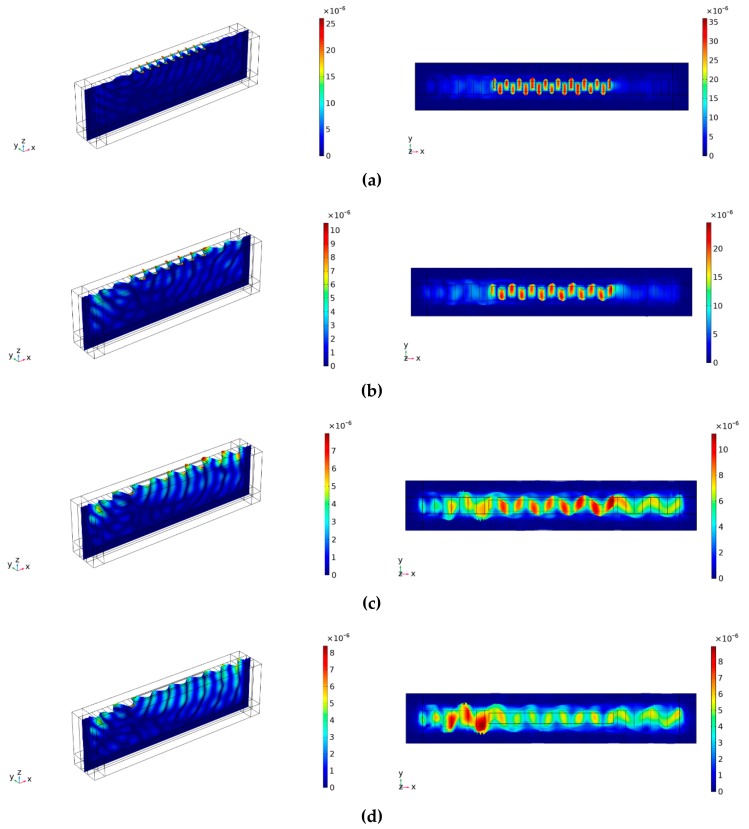
Snapshots of sectional views on a Y + 36° cut substrate with different thicknesses and materials (first column spatial displacement, second column displacement along the Y-direction) (**a**) 0.03 *λ*-thick gold wave-guide layer at 455 MHz; (**b**) 0.025 *λ*-thick platinum wave-guide layer at 421 MHz; (**c**) 0.035 *λ*-thick titanium wave-guide layer at 438 MHz; and (**d**) 0.03*λ*-thick silicon dioxide wave-guide layer at 449 MHz.

To further analyze the results obtained, sectional views with the strongest peak in different material layers are depicted in Figure 7, where wave patterns and wave-guiding statuses are clearly shown. It can be seen directly that the deposited layers all enhance the wave-guiding effect in comparison with the nonlayer situations. 

Among the layers, the Au layer successfully confines the wave on the surface significantly increasing the polarized displacements and converting the wave into a Love mode wave. The performance of the Pt layer is fine yet inferior to Au, where the predominant mode is still a Love wave, but some leakages can also be observed. In contrast to the former two, the situations of the Ti and SiO_2_ layers are worse, where the wave-guiding effect and conversion can be barely seen on the surface, showing a character of leaky SH-SAW [36]. In addition to the modes illustrated in Figure 7, it is very necessary to claim that the modes shown only in Figure 6 present the same type of waves as their respective counterparts on the same material layers. Thus, we concluded that Au layer with a thickness varying from 0.015 *λ* to 0.03 *λ* could be excellent for Y + 36° substrate to guide SH-SAWs near 433 MHz, while Pt, Ti, and SiO_2_ are not adequate for such work. 

In order to analyze the discrepancy of the wave-guiding layers, material properties relevant to acoustics were compared. Of the four materials, Au and Pt have a much lower shear velocity than quartz (*v_shear_* = 3310 m/s) and are more likely to guide and convert the resulting SH-SAWs. However, the Pt layer shows a different fluctuation tendency and wave transformation, which can be caused by its relatively higher shear velocity and relatively larger acoustic impedance. In terms of Ti and SiO_2_, much smaller acoustic impedance and higher shear velocity were present but there is no improvement. It is shown here that a lower shear velocity is apparently not the only important acoustic property for the guiding layers. A good combination of both shear velocity and acoustic impedance is the determinant. Maybe this is the reason that Au could successfully produce wave-guide effects.

### 4.3. Effect of External Applied Torque

To study the effect of external torque, the Y + 36° cut substrate with a 0.025 *λ*-thickness gold layer was chosen, based on the previous studies on the crystal cut effect and wave-guide layer effect. As mentioned at the beginning, when a torque is applied to the end of a shaft, sensors mounted along the ± 45° directions to the shaft axis are compressed and tensioned only. Therefore, external torques can be converted into normal stresses applied to the sensors. According to this, boundary loads varying from 0 to 63.5 MPa were assigned to the sides of sensors (as the boundary conditions described in Section 3), where the maximum value is calculated by Equation (1) using the parameter of 20 mm (shaft diameter) and 100 Nm (torque).

**Figure 8:**
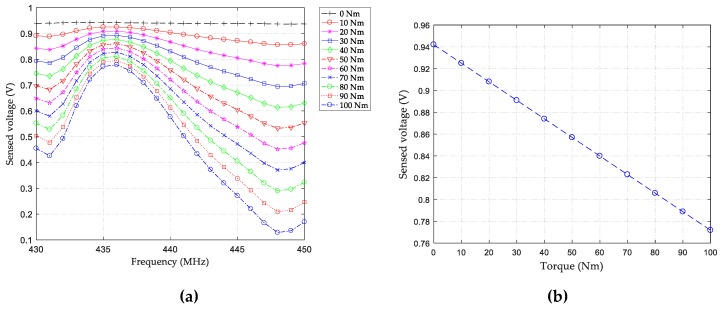
Input-output characteristic curves: (**a**) shifting voltage amplitudes against different frequencies and torques; (**b**) shifting voltage amplitudes against varying torques at 436 MHz.

Because the placed sensors operate under similar conditions, in the current work, we only examined the frequency-sensed voltage relationship of the sensor, which is fixed along the +45° direction to the shaft axis. The corresponding relationship of the other sensor can be checked using the same way as well. Figure 8a shows the relationship between frequency and sensed voltage when different torques are applied. It can be seen that, under the same frequency, the amplitudes of the sensed voltages decrease as the torque increases. This is caused by the total reverse piezoelectric effect resulting from the applied torque and the propagating SH-SAWs on the sensing IDTs. To explain this in detail, the second formula of Equation (3) is transformed into Equation (4), based on some electrical and mechanical considerations [38], wherein the electric displacement *D* satisfies the electrostatic equation for an insulator (quartz), so the divergence of electric displacement becomes zero: *D_i,i_* = 0; the strain *S* can be determined by mechanical displacement *u*, expressed as *S_ij_* = (*u_i,j_* + *u_j,i_*)/2; and the electric field *E* is derivable from electric potential *φ*, that is, *E_i_* = −*φ_,i_*. Hence, we can get:

(4) $$0=e_{ijk}u_{j,ki}-\varepsilon_{ij}\varphi_{,ij},$$

where the comma followed by an index denotes partial differentiation with respect to a space coordinate [38], and the constants are the same as the ones in Equation (3). 

To integrate this equation, the relationship between electric potential and displacement can be finally determined. Considering the strain along the −45° direction (directed toward the sensor) and the main displacement component of SH-SAWs along the *Y*-axis (the +45° direction, directed backward the sensor), it can be understood that the total displacement decreases when the torque increases, and hence, the total reverse piezoelectric effect on the sensing IDTs is weakened. As a result, a phenomenon of dropping sensing voltage can be observed when the torque increases. Taking into account the frequency of interest (near the ISM band) and frequency gap between two toque sensors, the responses of the sensor at 436 MHz were examined. Figure 8b plots the torque-sensed voltage curve, from which a perfect straight line is observed, indicating a good linearity of the torque sensor.

## 5. Conclusions

Torque sensors are widely used in modern industry and production to monitor momentum and ensure reliable torque transmissions. Although surface acoustic wave (SAW) torque sensors have been known and used for decades, the operational limits of such sensors have only recently become apparent. Featuring with a higher velocity and better power-handling capability, shear horizontal surface acoustic waves (SH-SAWs) are very suitable for torque sensor applications, having shown a great potential for the future. In order to design and develop such sensors, the propagation characteristics of SH-SAWs and sensing mechanism should be clearly understood. 

In this work, we proposed a numerical model for designing SH-SAW torque sensors, by which the effects of crystal cut, wave-guide layer, and external torque on the polarized displacement, wave guidance, and wave mode are investigated. Simulations, with the considerations of different Y-cut quartz substrates, different combinations of material layer and thickness, and varying torques, are conducted. It was shown that Y-cut quartz with an angle close to 36° can effectively generate SH-SAWs, while the cuts near −51° and at 90° are not adequate. The simulation results of the waveguiding effect show that a Y + 36° quartz substrate with an Au layer varying from *h_Au_/λ* = 0.02 to 0.03 thickness can be the most effective configuration for SH-SAW torque sensors operating around 433MHz, compared to other combinations using Pt, Ti, and SiO_2_. Meanwhile, a more general conclusion might be obtained, that is, to guide SH-SAWs, a successful wave-guiding layer should have a good combination of shear velocity and acoustic impedance, which could be a valuable reference for designing relevant sensors. Finally, based on the FEA SH-SAW torque sensor model, the relationship between the applied torque and response was examined, where a perfect linearity was obtained demonstrating a good performance of the sensor.

The proposed approach also allows extended studies for the torque sensor, for example, temperature effect. In our future research, a real SH-SAW torque sensor will be fabricated to demonstrate the proposed method and to more extensively compare the performance of SH-SAW and SAW torque sensors.

## Figures and Tables

**Table 1:** Part of material constants of gold, platinum, titanium, and silicon dioxide [39].

Material Constant	Au	Pt	Ti	SiO_2_
Young’s Modulus (GPa)	78	170	110	74
Poisson ratio	0.42	0.38	0.32	0.17
Density (×10^3^ kg/m^3^)	19.32	21.14	4.48	2.20
Shear velocity (m/s)	1200	1730	3100	2850
Acoustic impedance (×10^6^ kg/m^2^s)	51.20	63.42	27.33	12.67
